# Ten-words recall test: an effective tool to differentiate mild cognitive impairment from subjective cognitive decline

**DOI:** 10.3389/fpsyt.2024.1429934

**Published:** 2024-10-11

**Authors:** Hua Ren, Qiansen Feng, Lei Chen, Linlin Li, Jiayu Wang, Jiajing Wu, Li Dong, Tiejun Liu, Ziqi Wang

**Affiliations:** ^1^ The Clinical Hospital of Chengdu Brain Science Institute, Ministry of Education (MoE) Key Laboratory for NeuroInformation, School of Life Science and Technology, University of Electronic Science and Technology of China, The Fourth People's Hospital of Chengdu, Chengdu, China; ^2^ Nursing School of Zunyi Medical University, Guizhou, China; ^3^ Research Unit of NeuroInformation, Chinese Academy of Medical Sciences, Chengdu, China

**Keywords:** mild cognitive impairment, subjective cognitive decline, neuropsychological assessment, memory, word recall

## Abstract

**Introduction:**

Subjective cognitive decline (SCD) and mild cognitive impairment (MCI) are stages 2 and 3, respectively, of the Alzheimer’s continuum. The Alzheimer’s Disease Assessment Scale-Cognitive Subscale (ADAS-cog’s) ten-words recall test is a validated method for the early detection of cognitive impairment in Alzheimer’s disease. However, limited studies have investigated its ability to differentiate between SCD and MCI.

**Methods:**

203 participants with SCD and 62 participants with MCI underwent multiple neuropsychological assessments. The Mini-Mental State Examination (MMSE) and Montreal Cognitive Assessment-Basic (MOCA-B) served as brief global cognition tests. A binary logistic regression model was used to analyze the potential factors affecting MCI. The accuracy of the ten-words recall test was assessed using the area under the receiver operating characteristic (ROC) and the area under the curve (AUC).

**Results:**

The neuropsychological assessment revealed significant differences in the ten-words recall test scores between the SCD (median age 61 years; 70.44% female) and MCI (median age 64 years; 61.29% female) groups (*p* < 0.001), with the MCI group scoring the highest. Using a cut-off value of 3.15 for the ten-words recall test, sensitivity for distinguishing MCI from SCD reached 87%, while specificity stood at 61% (AUC 0.777, *p* < 0.001). DeLong’s test indicated no statistically significant difference in the ten-words recall test’s ability to distinguish between SCD and MCI compared to the total score of ADAS-Cog (AUC 0.833, *p*) and MMSE (AUC 0.784, *p* > 0.05). However, a significant difference was observed when compared to MoCA-B (AUC 0.973, *p* < 0.001). In the population with an education level of ≤ 9 years, the optimal cut-off value for the ten-words recall test was 3.15, yielding a sensitivity of 91% and a specificity of 45% (AUC = 0.674, *p* = 0.030). In the population with an education level > 9 years, the optimal cut-off value was 3.63, resulting in a sensitivity of 79% and a specificity of 71% (AUC = 0.785, *p* < 0.001).

**Discussion:**

The ten-words recall test from the ADAS-cog may detect MCI early owing to its simplicity and quick administration. It is an effective and convenient tool for rapidly identifying mild cognitive impairment.

## 1 Introduction

Alzheimer’s disease (AD) is suggested to have a clinically pre-symptomatic phase lasting 15–20 years (1), with the earliest clinically identifiable stage beginning with subjective cognitive decline (SCD) (2, 3). SCD is characterized by self-perceived cognitive impairment, particularly in memory, despite normal objective test results (4). As SCD advances, it transitions into mild cognitive impairment (MCI) (5, 6), wherein patients exhibit objective signs of memory impairment or cognitive decline but do not meet the criteria for significant functional impairment associated with dementia. SCD and MCI are stages 2 and 3, respectively, on the Alzheimer’s continuum (7). SCD patients may develop MCI after 10-15 years. However, not all people with SCD necessarily develop MCI or Alzheimer’s disease. Approximately 25% of patients with SCD may show progression to MCI (8). Identifying the transition from SCD to MCI is crucial for delaying dementia progression and enabling early intervention (9, 10).

Patients with SCD and MCI often undergo comprehensive cognitive screening to detect potential impairments while maintaining social functionality (11). However, this assessment process can be time-consuming and challenging in clinical settings, owing to the utilization of multiple tools and complex operations (12–14). AD often has impairment in multiple cognitive domains, including memory, language, visuospatial, attention and executive functioning, and general cognitive screening tools such as the Mini-Mental State Examination (MMSE) and the Montreal Cognitive Assessment (MOCA) are uselly used for AD screening (15). Memory complaints are common among patients with SCD and MCI, with studies indicating that amnestic MCI is more likely to progress to AD (16). Emphasizing memory assessment is crucial to enhance early detection of cognitive impairment. Commonly used memory scales include the Auditory Verbal Learning Test (AVLT), the Wechsler Memory Scale-Revised–Logical Memory test, the Rey Auditory Verbal Learning Test (RAVLT), the California Verbal Learning Test (CVLT), and the Hopkins Verbal Learning Test (HVLT), among others. These tests evaluate short-term memory, delayed recall, and recognition and provide insights into an individual’s memory abilities to varying degrees (17). However, their limitations include operational complexity and inconsistencies across clinical versions.

The Alzheimer’s Disease Assessment Scale (ADAS) was developed by Rosen and Mohs, with its cognitive component (ADAS-Cog) assessing memory, language, praxis, and attention abilities (18). Tailored to the cognitive characteristics of patients with AD, the ADAS is clinically valid and reliable for evaluating cognitive impairment levels (19). It is commonly used to assess the effectiveness of anti-dementia treatments and in longitudinal studies of cognitive decline (20), including research on MCI. However, the total ADAS score may lack sensitivity to MCI and frequently demonstrates a ‘ceiling effect.’ Further research is needed to investigate the importance of subitem scores in individual cognitive domain assessments. The ten-words test from the ADAS-Cog, which was chosen for the memory domain, has also been validated for detecting early cognitive decline (21). Chander et al. found that the ten-words recall task in the ADAS-Cog was used to assess the serial position effect (SPE) performance in relation to recency and primacy recall, which are associated with distinct clinical patterns of MCI pathophysiology. In the ten-words recall test, participants were shown a list of ten words and were told to read each word out aloud and memorize them. At the end of the exposure, participants were asked to repeat as many words as they could. The trial exposure and recall challenge was then repeated two more times, each using the same ten words in a pseudorandomized order. After approximately 5 minutes, participants were challenged to repeat as many of the ten words again (22). The test focusses on the immediate recall and delayed recall, which are highly associated with cognitive impairment.

Therefore, this study aims to investigate the discrimination power of the ADAS-cog’s ten-words recall test for detecting MCI versus SCD, which may offer an early and cost-effective SCD tool for screening cognitive function in the aging population.

## 2 Materials and methods

### 2.1 Participants

Between April and October 2023, older individuals aged 50–80 years were recruited from community and memory clinics for this study. Written informed consent was obtained from the participants or their families, and the study received approval from the Medical Ethics Committee of the Fourth People’s Hospital of Chengdu (approval number: 2022), Ethics Review No. (76).

Participants underwent a comprehensive assessment, including a general questionnaire survey, physical examination, laboratory tests, and cognitive assessments. Laboratory tests included routine blood tests, folate and vitamin B12 levels measurements, liver and kidney function assessments, lipid profile analysis, blood sugar evaluation, thyroid function tests, and resting-state MRI brain scans to rule out cognitive impairments caused by physical illnesses. Cognitive assessments comprised the ADAS-Cog, MMSE, Montreal Cognitive Assessment-Basic (MOCA-B), Activities of Daily Living (ADL), and Clinical Dementia Rating (CDR).

Inclusion criteria were as follows: (1) elementary school education or higher, with no limitations based on marital status or occupation; (2) sufficient vision and hearing to complete the study; and (3) preserved general daily living abilities with voluntary participation in the examination and signing of an informed consent form. Exclusion criteria included neurological (i.e., epilepsy or ongoing spells suggestive of seizures, a history of stroke, traumatic brain injury with loss of consciousness, meningitis/encephalitis, brain tumor, or brain surgery) or psychiatric diseases (i.e., drug or alcohol abuse, chronic psychoactive drugs used), as well as current use of anti-seizure medications, benzodiazepines, sleep aids, or bupropion.

The diagnosis of MCI was based on the diagnosis standard proposed by Petersen (23): (1) self- or informant-reported cognitive complaint; (2) objective memory impairment; (3) preserved independence in functional abilities (ADL assessment is normal); and (4) absence of dementia. Cognitive function criteria were as follows: MMSE: ≥ 26 points for university education, ≥ 24 for secondary education, and ≥ 20 for primary education; MOCA-B: ≤ 19 points for primary education, ≤ 22 for secondary, and ≤ 24 for university and above; CDR ≤ 0.5 points; ADL: < 23 points for those under 75 years and < 25 points for those 75 years and above; Hachinski Ischemia Scale (HIS): ≤ 4 points; and HAMD-17: < 17 points.

The diagnosis of SCD referenced the criteria of the Subjective Cognitive Decline Initiative (SCD-I) in 2014 (24): (1) The individual subjectively feels a significant memory decline; (2) This feeling has arisen within the last 5 years; (3) Age ≥ 50 years; (4) Concern about the memory decline; (5) Memory perceived to be worse compared to that of peers; (6) CDR = 0 points; (7) ADL < 23 points for those under 75 years, and < 25 points for those 75 years and above; (8) HIS: ≤ 4 points; and (9) HAMD: < 17 points.

Following the inclusion and exclusion criteria, 62 patients with MCI and 203 with SCD were included. Prior to the study, all neuropsychological assessment researchers underwent standardized training and met consistency criteria. The ultimate diagnosis of all the participants was assessed and confirmed by another attending physician or an associate chief physician. Two physicians holding senior professional titles deliberated and resolved any contested diagnoses.

### 2.2 Research tools

ADAS-cog (25): The ADAS-cog comprises 12 tasks, including word recall, naming, following commands, constructional praxis, ideational praxis, orientation, word recognition, instruction remembering, spoken language ability, word-finding difficulty, comprehension, and attention. Scores range from 0 to 70, with higher scores indicating more severe cognitive impairment.

MMSE (26): The MMSE is a widely used cognitive impairment screening tool globally. It comprises 19 items assessing memory, calculation, attention, language abilities, memory, calculation and visual-spatial skills. Scores range from 0 to 30, with higher scores indicating better cognitive function.

MOCA-B (27): The basic version of the MOCA includes visual-spatial abilities, executive functions, naming, memory, attention, language, abstraction, and orientation. The total possible score is 30, with higher scores indicating better cognitive function.

The CDR (28): clinically grades the severity of cognitive and social functioning impairments in older individuals (primarily patients with dementia) through semi-structured interviews with the participant and an informed caregiver. It assesses six domains (memory, judgment and problem-solving, orientation, personal care, home and hobbies, and community affairs) using a grading scale of 0–3, with higher scores indicating more severe dementia.

ADL (29): ADL comprises 20 items, encompassing physical self-care activities such as eating, dressing, grooming, toileting, and walking, as well as instrumental activities of daily living such as housework, shopping, using the telephone, and managing finances. Higher scores indicate a more pronounced functional decline on a 4-point scale (1–4).

HIS (30): The HIS is a simple screening tool for vascular dementia designed to differentiate between vascular dementia and AD. It comprises 13 items, with a maximum score of 18. A total score of ≤ 4 points suggests Alzheimer’s disease, while ≥ 7 points indicates vascular dementia.

HAMD (31): This scale includes depression, feelings of guilt, suicidal thoughts, difficulty falling asleep, non-restorative sleep, early morning awakening, loss of interest, sluggish thinking, agitation, mental anxiety, somatic symptoms, gastrointestinal symptoms, genital symptoms, hypochondriasis, weight loss, and insights. Rated on a 5-point scale, higher scores indicate more severe depression.

### 2.3 Statistical methods

All data were inputted into SPSS version 26.0. Group disparities were compared, with categorical variables delineated by absolute values and composition ratios using the χ^2^ test. Continuous variables not conforming to a normal distribution were presented as medians (interquartile ranges) and evaluated via the Wilcoxon rank-sum test. Spearman’s rank correlation coefficients were computed between the scores of the ten-words recall test, ADAS-Cog, MMSE, and MOCA. The Spearman’s rank correlation test was utilized to ascertain the correlation and consistency among the scores of these four scales. Higher consistency suggests that the four scales have a better convergent validity of cognitive assessment. Receiver operating characteristic (ROC) curves were constructed for the four scales to discriminate between MCI and SCD, obtaining the area under the curve (AUC) along with the cut-off value, sensitivity, and specificity of the ten-words recall test at its maximum AUC. The accuracy of the test was evaluated by the AUC, whereby AUC = 0.5 meant no diagnostic ability and AUC = 1 meant perfect diagnostic ability (32). DeLong’s test was employed to conduct a differential analysis of the area under the ROC curve (AUC) to assess the discriminative ability of the ten-words recall test between MCI and SCD. Differences were considered statistically significant at a significance level of *p* < 0.05.

## 3 Results

### 3.1 General demographic data and overall cognitive status comparison

A total of 265 valid samples were collected, comprising 62 from the MCI group and 203 from the SCD group. There were no statistically significant differences in sex or age between the two groups (*p* = 0.175 and *p* = 0.168, respectively). Significant differences were observed in the years of education (*p* = 0.002), MMSE (*p* < 0.001), MOCA (*p* < 0.001), ADAS-Cog total score (*p* < 0.001), and ten-words recall test score (*p* < 0.001). The MMSE and MOCA-B total scores in the MCI group were lower than those in the SCD group, while the ADAS-Cog total scores and ten-words recall test scores were higher in the MCI group (**Table 1**).

**Table 1:** Participant demographics.

Variable	Group		Statistical test	Value	*P* value
	SCD (n = 203)	MCI (n = 62)			
**Sex**			χ^2^	1.84	0.175
Male	60 (29.56%)	24 (38.71%)			
Female	143 (70.44%)	38 (61.29%)			
**Age (years)**	61 (54,68)	64 (57,68)	Wilcoxon W	26272	0.168
**Education (years)**	13 (11,16)	12 (8,15)	Wilcoxon W	6586.5	0.002
**Ten-words recall test (points)**	3 (2,4)	4.33 (3.67,5)	Wilcoxon W	23515.5	<0.001
**ADAS-Cog (points)**	6 (4.33,8.67)	11.33 (8.17,15.08)	Wilcoxon W	22813	<0.001
**MMSE (points)**	29 (28,30)	27 (25,28)	Wilcoxon W	4667	<0.001
**MoCA-B (points)**	26 (25,27)	21 (19,22)	Wilcoxon W	2294.5	<0.001

Categorical variables are described using absolute values and composition ratios, with differences between groups compared using the χ2 test. Continuous variables (age, education, and test scores) not conforming to a normal distribution are presented as medians (interquartile ranges) and analyzed using the Wilcoxon rank-sum test. SCD, subjective cognitive decline; MCI, mild cognitive impairment.

### 3.2 Comparison of ten-words recall test scores

**Figure 1:**
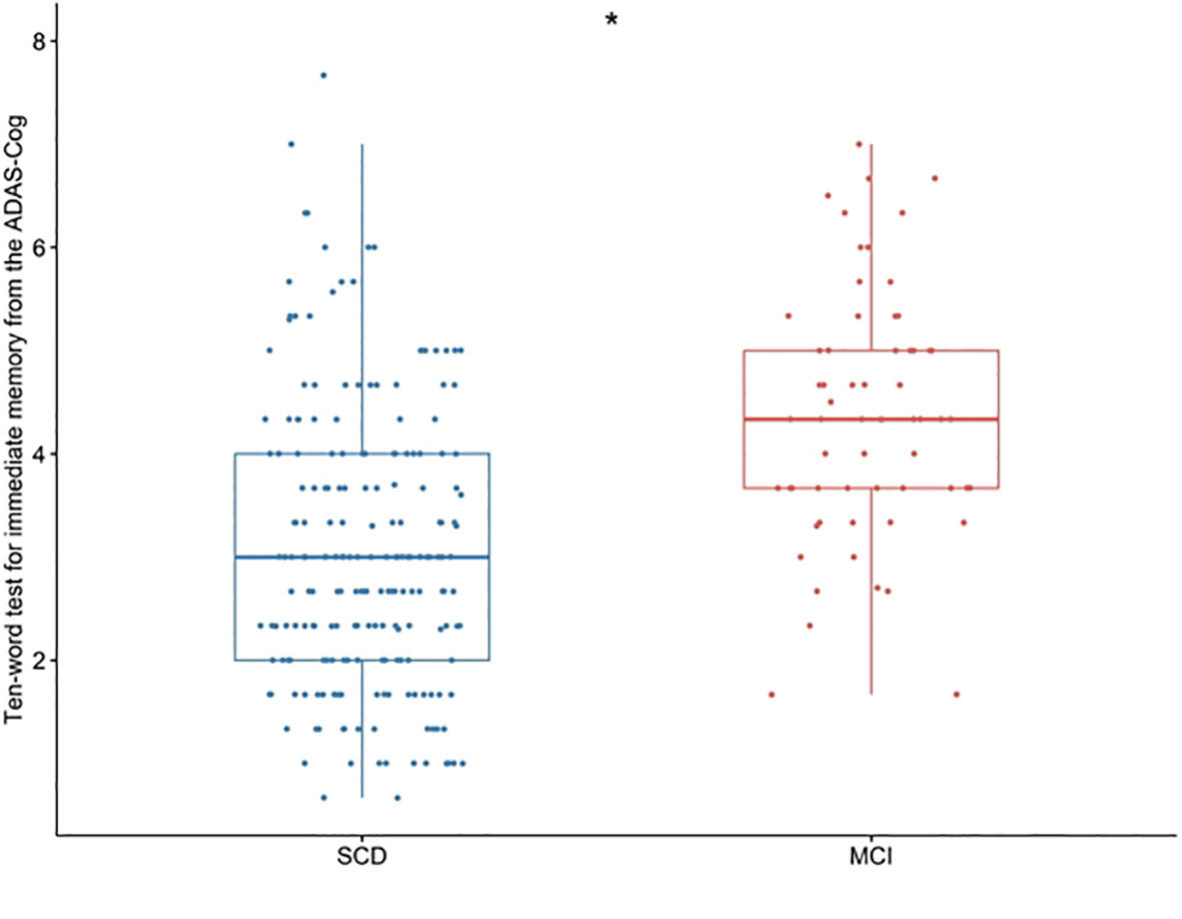
Differences in the ten-words recall test for immediate memory from the ADAS-Cog among groups were tested using the Wilcoxon W test. *p < 0.001. SCD, subjective cognitive decline; MCI, mild cognitive impairment. ADAS-Cog, Alzheimer’s Disease Assessment Scale-Cognitive section.

Scatter plots were utilized to visualize the ten-words recall test scores of the two groups. A significant difference between the SCD and MCI groups was observed, with the MCI group displaying higher scores on the ten-words recall test (*p* < 0.001; **Figure 1**).

### 3.3 Correlation analysis between ten-words recall test and ADAS-Cog total score, MMSE, and MoCA-B

The results revealed Spearman’s rank correlation coefficients between the ten-words recall test and the ADAS-Cog total score, MMSE, and MOCA-B as 0.692, 0.393, and 0.461, respectively, signifying significant correlations (*p* < 0.01) (**Table 2**).

**Table 2:** Correlation coefficient matrix of the scores from different scales.

	Ten-words recall test	ADAS-Cog	MMSE	MoCA-B
**Ten-words recall test**	1	0.692**	− 0.393**	− 0.461**
**ADAS-Cog**	0.692**	1	− 0.535**	− 0.533**
**MMSE**	− 0.393**	− 0.535**	1	0.524**
**MoCA-B**	− 0.461**	− 0.533**	0.524**	1

Because the scoring results of each scale did not conform to a normal distribution, Spearman’s rank correlation coefficients were calculated first, followed by Spearman’s rank correlation test for correlation analysis. The Spearman’s rank correlation coefficients between the Different Scales. ** P < 0.01. ADAS-cog, Alzheimer’s Disease Assessment Scale-Cognitive Subscale; MMSE, Mini-Mental State Examination; MOCA-B, Montreal Cognitive Assessment-Basic.

### 3.4 Discriminative power of ten-words recall test for SCD and MCI

**Figure 2:**
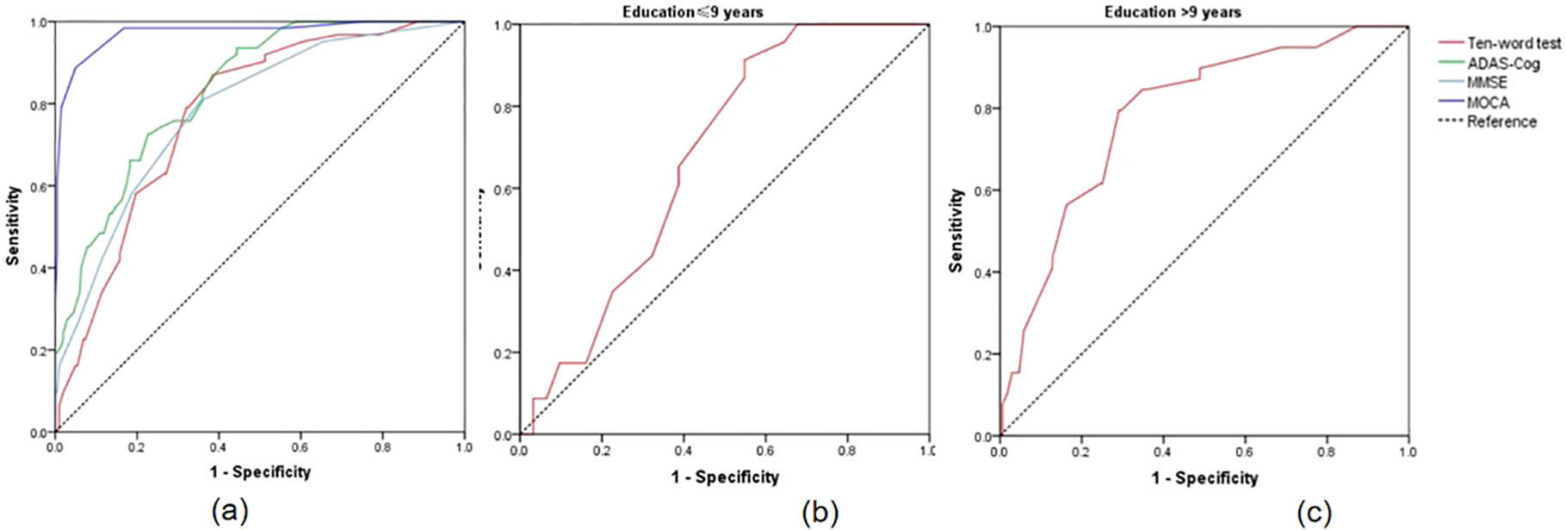
**(A)** ROC curves for the discrimination between SCD and MCI; **(B)** ROC curves for distinguishing MCI with educational levels ≤9 years using ten-words test; **(C)** ROC curves for distinguishing MCI with educational levels ≤9 years using ten-words test. SCD, subjective cognitive decline; MCI, mild cognitive impairment; ROC, receiver-operating characteristic.

The results indicated that when the cut-off value of the ten-words recall test was set at 3.15, the AUC was 0.777, with a sensitivity of 87% and specificity of 61%, demonstrating an excellent discriminative ability for distinguishing MCI from SCD (*p* < 0.001) (**Table 3**). DeLong’s test indicated that the difference between the ten-words recall test and the ADAS-Cog total score was not statistically significant (Z = -1.92, *p* = 0.055; *p* = 0.843, Z = -0.20, respectively). However, a significant difference was observed compared to the MoCA-B (AUC, 0.973) (*p* < 0.001) (**Table 4** and **Figure 2A**).

**Table 3:** Cut-off points and diagnostic utility of the ten-words recall test for immediate memory from the ADAS-Cog to discriminate between SCD and MCI groups.

Variables	Cut-off value	Sensitivity	Specificity	AUC	SE	Value	95% CI	
							LL	UL
**Ten-words recall test(points)**	3.15	0.87	0.61	0.777	0.031	< 0.001	0.717	0.837
**ADAS-Cog (points)**	9.00	0.73	0.77	0.833	0.026	< 0.001	0.781	0.884
**MMSE (points)**	28.49	0.81	0.65	0.784	0.032	< 0.001	0.721	0.848
**MoCA-B (points)**	23.49	0.89	0.95	0.973	0.012	< 0.001	0.949	0.996

SCD, subjective cognitive decline; MCI, mild cognitive impairment; ADAS-cog, Alzheimer’s Disease Assessment Scale-Cognitive Subscale; MMSE, Mini-Mental State Examination; MOCA-B, Montreal Cognitive Assessment-Basic; SE, standard error; 95% CI, 95% confidence interval; LL, lower limit 95% confidence interval; UL, upper limit 95% confidence interval; AUC, area under the curve.

**Table 4:** DeLong’s test for the AUC of the ROC curves of each scale.

	ADAS-Cog	MMSE	MoCA-B
***Z* value**	−1.92	− 0.20	− 6.13
***P* value**	0.055	0.843	< 0.001

AUC, the area under the curve; ROC, receiver-operating characteristic; ADAS-cog, Alzheimer’s Disease Assessment Scale-Cognitive Subscale; MMSE, Mini-Mental State Examination; MOCA-B, Montreal Cognitive Assessment-Basic.

### 3.5 Subgroup analysis

Statistically significant differences in educational levels between the two groups were evident (**Table 1**). Further subgroup analysis based on years of education was conducted to explore the optimal cut-off value of the ten-words recall test for individuals with different educational backgrounds. ROC curve analysis was employed to determine the cut-off values for the ten-words recall test in different education level groups, calculating the AUC, where a larger AUC indicates better discriminative power. The results revealed that the optimal cut-off value was 3.15 points for the population with an education level of ≤ 9 years, demonstrating a sensitivity of 91% and specificity of 45% (AUC = 0.674, *p* = 0.030). For those with an education level of > 9 years, the optimal cut-off value was 3.63 points, with a sensitivity of 79% and specificity of 71% (AUC = 0.785, *p* < 0.001) (**Table 5**). The area under the curve indicates that patients with higher educational levels exhibit a better discriminative ability to identify MCI using the ten-words recall test for memory assessment (**Figures 2B, C**).

**Table 5:** The optimal cut-off value and AUC for different educational backgrounds.

Education	Optimal cut-off	Sensitivity	AUC	SE	P value	95% CI	
						LL	UL
**≤9 years**	3.15	0.91	0.674	0.030	0.030	0.532	0.816
**>9 years**	3.63	0.79	0.785	0.038	<0.001	0.710	0.860

LL, lower limit 95% confidence interval; UL, upper limit 95% confidence interval; AUC, the area under the curve, SE, standard error, CI, confidence interval.

## 4 Discussion

SCD and MCI represent precursor stages of AD that can serve as targets for early treatment. However, efficient and convenient tools for their identification in clinical settings are lacking. In this study, we found that the MCI group had higher scores on the ADAS-Cog total score and ten-words recall test than the SCD group, indicating more severe cognitive impairment. Spearman’s correlation analyses revealed that the ten-words recall test from the ADAS-cog demonstrated convergent validity with the MMSE and MOCA-B total scores, suggesting good parallel validity. This indicates that it can achieve a similar effect as a comprehensive neuropsychological evaluation in identifying cognitive impairment.

As widely recognized, neuropsychological assessments are pivotal in identifying early cognitive alterations suggestive of MCI and SCD (33, 34). MCI and SCD lack specific screening tools and primarily utilize assessment scales designed to assess dementia (35). In this context, two primary types of cognitive assessments are employed: general cognitive screening tools such as the MMSE and comprehensive evaluations of cognitive domains such as the MoCA (36). They are widely used for dementia screening and diagnosis but have limited sensitivity to mild cognitive impairment. These tests cover a broad range of cognitive functions but lack depth (37). In contrast, domain-specific cognitive assessments focus on specific cognitive domains. For instance, the Shape Trails Test primarily assesses executive function, the Clock Drawing Test evaluates visuospatial abilities, the Boston Naming Test and Animal Fluency Test targets language abilities, and the Auditory Verbal Learning Test focuses on memory (38). Based on the results of these tests, slight abnormalities suggested MCI, while the absence of abnormalities suggested SCD. These domain-specific tools offer comprehensive evaluations of specific cognitive areas and, when used together, can effectively identify mild cognitive impairment. However, they are less conducive to rapid screening and widespread use because of the multitude of tests and requirements for professional neuropsychological knowledge. An ideal cognitive screening tool should possess several key attributes: it should be quick to administer (typically < 5 min), easy to use (without requiring specialized equipment), and well-received by patients. The ten-words recall test in our study simplifies the process, is straightforward to administer, and demands minimal auxiliary equipment and computation time.

Furthermore, a good assessment tool should have clearly defined cut-off values and established sensitivity, reliability, and validity (39). These attributes guarantee the tool’s effectiveness across different settings, from clinical environments to potentially broader community-based screenings (40). In our study, the MCI group demonstrated higher scores on the ADAS-cog and ten-words recall test but lower scores on the MMSE and MOCA than the SCD group. This suggests that patients with MCI exhibit more pronounced overall cognitive impairment and memory deficits compared to those with SCD, aligning with clinical observations. Furthermore, our study precisely defined the cut-off value for the ten-words recall test through ROC curve analysis (41, 42). Setting the cut-off value at 3.15 (AUC=0.777), the test’s capacity to differentiate MCI was comparable to that of the MMSE (cut-off value=28.49; AUC=0.784) and ADAS-cog (cut-off value=9.00; AUC=0.833). This discovery emphasizes the efficacy of the ten-words recall test as a rapid and effective tool for cognitive screening, especially in discerning between MCI and SCD. Validating this cut-off value enhances the practicality of the ten-words recall test in diverse clinical and community settings, providing a swift and efficient method for early cognitive decline detection. Although the cut-off value at 3.15 has the best sensitivity and specificity to help identify MCI, many false positives or false negatives still exist. A clear diagnosis also requires a combination of interviews with clinicians and other auxiliary methods.

Moreover, this study revealed variations in the cut-off values of the ten-words recall test among different education level groups, indicating distinct levels of discrimination. Participants with higher education levels exhibited enhanced capacity to differentiate between MCI and SCD using the ten-words recall test for memory assessment. This finding aligns with the conventional practice in cognitive assessment tools such as the MMSE and MOCA, where cut-off values are adjusted according to educational attainment levels (43). The observed variation in cut-off values can be attributed to differences in cognitive reserves among individuals with diverse educational backgrounds. Previous cognitive reserves can compensate for and conceal cognitive impairments (44). Epidemiological studies suggest that lifelong experiences, such as education and occupational accomplishments, contribute to an individual’s cognitive reserve (45). Research indicates that a robust cognitive reserve is a protective factor against cognitive impairment, allowing individuals to better tolerate changes in cognitive levels while maintaining functionality (46). Individuals with higher educational levels may retain relatively intact abilities in cognitive domains such as computation, executive function, language ability, and spatial structure (47). This can lead to higher overall scores in comprehensive cognitive assessments, potentially concealing impairments in short-term memory and leading to missed diagnoses. Therefore, the ten-words recall test was higher in populations with greater educational attainment, where memory impairment was more pronounced. This finding is consistent with previous research indicating that memory impairment is the most common complaint in patients experiencing early cognitive decline (15, 16).

This study’s limitations include the absence of longitudinal research and follow-up of participants in each group. Clinical scale assessments are susceptible to subjective influences, and this study did not integrate multidimensional validation with objective evidence, such as biomarkers or neuroimaging. Future studies should incorporate longitudinal follow-up to track changes in cognitive function over time and integrate multidimensional analysis and validation incorporating biomarkers and neuroimaging data. These comprehensive approaches will advance our comprehension of cognitive impairment progression and the efficacy of early screening tools (47).

In summary, early cognitive screening is pivotal in the prevention and treatment of dementia, with cognitive assessment serving as the foundation. Employing simple and rapid tools can offer substantial advantages in effectively conducting widespread early cognitive screening. Memory impairment is the most common complaint in MCI and SCD. Therefore, rapid screening targeting memory should be emphasized. This study revealed that the ten-words recall test can objectively assess short-term memory levels. It is simple to operate and quick to administer and demonstrates good discriminative ability between individuals with MCI and SCD. It is an effective and convenient tool for swiftly identifying mild cognitive impairment.

## Data Availability

The original contributions presented in the study are included in the article/supplementary material. Further inquiries can be directed to the corresponding author.
